# Associations of dietary quality and physical activity with glycemic control in patients with type 2 diabetes: the moderating role of trait mindfulness

**DOI:** 10.3389/fendo.2026.1910587

**Published:** 2026-07-22

**Authors:** Jiaxin Li, Daowen Zhang, Weiwei Zhu, Huijun Zhang, Cancan Liu, Fang Zhang, Mengmeng Yan, Kuanlu Fan

**Affiliations:** Department of Endocrinology, The Second Affiliated Hospital of Xuzhou Medical University, Xuzhou, China

**Keywords:** diet, exercise, glycemic control, trait mindfulness, type 2 diabetes

## Abstract

**Background:**

Dietary quality and physical activity are fundamental components of glycemic management in patients with type 2 diabetes mellitus (T2DM). However, glycemic responses to lifestyle behaviors may vary across individuals, suggesting a potential role of psychological self-regulation traits. This study aimed to assess the associations of dietary quality and physical activity with glycemic indicators among hospitalized patients with T2DM and to test whether the Act with Awareness dimension of trait mindfulness moderated these associations using interaction models.

**Methods:**

This cross-sectional study included 400 hospitalized patients with T2DM at the Second Affiliated Hospital of Xuzhou Medical University between December 2023 and June 2025. Dietary quality was assessed using a structured dietary score, and physical activity was evaluated according to self-reported weekly exercise duration. Trait mindfulness was measured using the Five Facet Mindfulness Questionnaire. The primary outcomes were glycated hemoglobin (HbA1c) and fasting blood glucose (FBG), both of which were natural log-transformed before regression analyses. Multivariable linear regression models were used to estimate the associations of dietary quality and physical activity with log-transformed HbA1c and FBG. Multiplicative interaction terms were then included to test the moderating role of Act with Awareness, with adjustment for demographic characteristics, lifestyle factors, BMI, diabetes duration, sleep, comorbidities, medication use, and insulin use.

**Results:**

In fully adjusted models, higher dietary scores were associated with lower log-transformed HbA1c (β = -0.019, 95% CI: -0.032 to -0.005; P = 0.008) and FBG (β = -0.023, 95% CI: -0.042 to -0.003; P = 0.022). Physical activity scores were negatively associated with HbA1c (β = -0.002, 95% CI: -0.004 to 0.000; P = 0.034), but not with FBG. Significant interactions were observed between Act with Awareness and dietary score for both HbA1c and FBG, and between Act with Awareness and physical activity score for HbA1c.

**Conclusion:**

Better dietary quality and higher physical activity were associated with more favorable glycemic indicators among hospitalized patients with T2DM. Act with Awareness may modify the strength of these associations. Given the cross-sectional design, these findings should be interpreted as observational associations rather than causal relationships.

## Introduction

Type 2 diabetes mellitus (T2DM) is one of the chronic metabolic diseases contributing most substantially to the global disease burden (1). With population aging, the increasing prevalence of obesity, and ongoing lifestyle changes, the number of individuals affected by T2DM continues to rise. Chronic complications resulting from suboptimal glycemic control, including cardiovascular disease, nephropathy, retinopathy, and neuropathy, further increase the long-term burden on both patients and healthcare systems (2). Therefore, optimizing lifestyle management beyond pharmacological treatment remains a critical component of comprehensive T2DM prevention and care.

Dietary control and regular physical activity are fundamental strategies in T2DM management (3, 4). Higher dietary quality may improve the overall structure of energy intake, reduce consumption of refined carbohydrates and saturated fats, and increase intake of dietary fiber and high-quality protein, thereby contributing to metabolic processes related to insulin sensitivity, weight management, and postprandial glucose fluctuations (5, 6). Physical activity, in turn, may be associated with better long-term glycemic control by promoting skeletal muscle glucose uptake, improving insulin resistance, modulating body fat distribution, and reducing chronic low-grade inflammation (7, 8). Previous studies have consistently suggested that healthier dietary patterns and higher levels of physical activity are associated with lower HbA1c levels, which is why they are recommended as core lifestyle strategies in major diabetes management guidelines (9).

However, in real world clinical practice, a substantial gap remains between knowing lifestyle recommendations and adhering to them over the long term. Many patients with T2DM show considerable individual variability in glycemic control despite receiving similar dietary and exercise guidance (10). Such variability may be related not only to conventional factors, such as age, sex, diabetes duration, obesity status, and medication use, but also to individual differences in psychological self-regulation and behavioral implementation (11). Dietary control and exercise management are not merely external behavioral exposures; rather, they require patients to continuously make choices, inhibit impulses, maintain goals, and adjust behaviors in daily life (12, 13). Therefore, explaining differences in glycemic control solely on the basis of dietary intake or exercise duration may not fully capture the complexity of lifestyle management in T2DM.

In recent years, mindfulness has received increasing attention in the field of chronic disease behavioral management as a psychological trait related to self-regulation (14). Mindfulness generally refers to the ability to attend to present-moment experiences in an open, accepting, and non-automatic manner (15). The Five Facet Mindfulness Questionnaire conceptualizes mindfulness across five dimensions: Observe, Describe, Act with Awareness, Non-judging, and Non-reactivity. Among these, Act with Awareness primarily reflects the capacity to maintain attentional focus during daily activities, reduce automatic responses, and engage consciously in current behavior. Compared with general emotional regulation or relaxation states, Act with Awareness is more directly related to the execution of goal-directed behaviors and may therefore be more closely linked to dietary control and exercise adherence (16).

To date, the associations of diet and physical activity with glycemic control in patients with type 2 diabetes mellitus (T2DM) have been extensively investigated. However, most previous studies have primarily focused on the direct relationships between lifestyle behaviors and metabolic outcomes. In contrast, relatively limited attention has been paid to whether psychological traits may modify the associations between lifestyle behaviors and glycemic control. This issue may be particularly relevant in hospitalized patients with T2DM, among whom dietary quality, physical activity, trait mindfulness, and glycemic indicators such as HbA1c and fasting blood glucose (FBG) may be closely interrelated. Moreover, previous mindfulness-related studies have mainly focused on the effects of mindfulness-based interventions on psychological distress or metabolic outcomes, whereas fewer studies have examined the potential moderating role of trait mindfulness in everyday dietary and physical activity behaviors.

Therefore, the present study aimed to investigate the associations of dietary quality and physical activity level with glycemic control indicators among hospitalized patients with T2DM. We further examined whether trait mindfulness, interacted with dietary quality and physical activity in relation to HbA1c and FBG. By integrating lifestyle behaviors with psychological self-regulation traits, this study sought to provide observational evidence for understanding individual differences in glycemic control among patients with T2DM and to inform future intervention studies that combine dietary, physical activity, and psychological behavioral management strategies.

## Methods

### Study population

This hospital-based cross-sectional study was conducted in the Department of Endocrinology at the Second Affiliated Hospital of Xuzhou Medical University between December 2023 and June 2025. A consecutive sampling strategy was used. During the study period, hospitalized patients with confirmed T2DM were screened in chronological order according to the eligibility criteria. Eligible patients who were clinically stable, able to complete questionnaire-based assessments, and willing to provide written informed consent were invited to participate. During the study period, 423 hospitalized patients with confirmed T2DM were initially screened. After excluding 23 patients with missing data on dietary quality, physical activity, or related covariates, 400 hospitalized patients with complete data were included in the final analysis. The inclusion criteria were (1): diagnosis of T2DM according to the Chinese Guidelines for the Prevention and Treatment of Type 2 Diabetes (17); and (2) age ≥18 years. Exclusion criteria were (1): acute complications of diabetes (e.g., hyperosmolar nonketotic coma, hypoglycemic coma, diabetic ketoacidosis) or severe infections (2); other severe acute or chronic organ or systemic diseases (3); infectious diseases such as hepatitis or tuberculosis, or any malignancy (4); severe psychiatric disorders, significant visual or hearing impairment; and (5) prediabetes, type 1 diabetes, gestational diabetes, or other forms of diabetes. The study protocol complied with national ethical standards and was approved by the Biomedical Ethics Committee of Xuzhou Mining Group General Hospital (the Second Affiliated Hospital of Xuzhou Medical University; Approval No. [2024]081301). All participants provided written informed consent prior to participation in the study.

### General information questionnaire

#### General information collection

Within 48 hours of hospital admission, trained investigators collected participants’ demographic and clinical information using a standardized questionnaire. Data included age, sex, education level (elementary school or below, junior high school, college or above), duration of diabetes (<1 year, 1–5 years, 5–10 years, ≥10 years), smoking history, alcohol consumption, body mass index (BMI), presence of hypertension or cardiovascular disease (CVD), medication intensity (low, moderate, high), insulin use (no/yes), average nighttime sleep duration, and average daytime nap duration. Glucose-lowering medication intensity was categorized according to the number of non-insulin glucose-lowering medication classes used before hospital admission. Low intensity was defined as the use of one non-insulin glucose-lowering medication class, moderate intensity as the use of two non-insulin glucose-lowering medication classes, and high intensity as the use of three or more non-insulin glucose-lowering medication classes. Insulin use was recorded separately as yes or no and was additionally adjusted for in the multivariable models.

#### Laboratory tests

Fasting venous blood samples were obtained after at least 8 hours of overnight fasting. Laboratory analyses measured fasting blood glucose (FBG), glycated hemoglobin (HbA1c), C-reactive protein (CRP), triglycerides (TG), total cholesterol (TC), high-density lipoprotein cholesterol (HDL-C), and low-density lipoprotein cholesterol (LDL-C) using standardized biochemical methods in the hospital’s central laboratory.

### Survey tools

Mindfulness was assessed using the validated Chinese version of the Five Facet Mindfulness Questionnaire (FFMQ), which comprises 39 items across five dimensions (18): Observe (8 items), Describe (8 items), Act with Awareness (8 items), Non-reactivity to Inner Experience (7 items), and Non-judging of Inner Experience (8 items). Each item is rated on a 5-point Likert scale ranging from 1 (“never or rarely true”) to 5 (“very often or always true”). Total mindfulness scores are calculated by summing all item scores, with higher scores indicating greater mindfulness. In the present sample, the Chinese version of the FFMQ demonstrated good internal consistency. Cronbach’s α was 0.879 for the total scale, and 0.913, 0.800, 0.912, 0.881, and 0.892 for the observing, describing, acting with awareness, non-judging, and non-reactivity subscales, respectively.

### Assessment of dietary quality

Dietary quality was assessed using an investigator-developed structured dietary quality score. This score was constructed with reference to the dietary component of the Life’s Essential 8 framework and the Dietary Guidelines for Chinese Residents, and was adapted to reflect commonly recommended healthy dietary behaviors in Chinese patients with T2DM (19, 20). The score was intended to capture overall dietary quality rather than precise nutrient intake.

The questionnaire included 16 dietary components: cooking oil, vegetables, fruits, salt intake, red and processed meat, fish and seafood, poultry, dairy products, butter or cream, legumes, whole grains, sweets and pastries, nuts, sugar-sweetened beverages, fast food, and alcohol intake. Each component was scored as 1 if the predefined healthy dietary criterion was met and 0 otherwise. The detailed scoring criteria for all 16 components are presented in **Supplementary Table 1**. The total raw score ranged from 0 to 16, with higher scores indicating better dietary quality. For descriptive and regression analyses, the raw dietary score was used as a continuous variable. In supplementary categorical analyses, dietary quality was further grouped according to the sample distribution.

Because this dietary quality score was a criterion-based composite index derived from established dietary recommendations rather than a psychometric scale designed to measure a single latent construct, internal consistency coefficients such as Cronbach’s α were not calculated. Content validity was supported by constructing the score according to established dietary recommendations from the Life’s Essential 8 framework and the Dietary Guidelines for Chinese Residents. However, the score has not been externally validated in an independent population, and this limitation has been acknowledged in the revised Discussion.

### Assessment of physical activity

Physical activity was assessed using an investigator-developed duration-based physical activity score. Physical activity information was collected by trained investigators using a structured questionnaire item. Participants were asked: “How long do you exercise per week?” Before answering this question, participants were provided with examples of moderate- and vigorous-intensity activities, such as housework, brisk walking, cycling, dancing, table tennis, Tai Chi, yoga, aerobics, stair climbing, jogging, basketball, football, rope skipping, and swimming, to help them understand the scope of physical activity. However, the questionnaire only recorded total weekly exercise duration, and exercise type and intensity category were not separately collected or scored.

The reported weekly exercise duration was converted into a duration-based score with reference to commonly recommended physical activity targets for adults and patients with T2DM, particularly the recommendation of at least 150 minutes of physical activity per week. Participants reporting ≥150 minutes of exercise per week were assigned 100 points; 120–149 minutes, 90 points; 90–119 minutes, 80 points; 60–89 minutes, 60 points; 30–59 minutes, 40 points; 1–29 minutes, 20 points; and 0 minutes, 0 points. Higher scores indicated longer weekly exercise duration.

Because this scoring method was developed for the present study and was based only on self-reported weekly exercise duration, it did not distinguish exercise type, intensity, frequency, timing, sedentary behavior, or long-term adherence. Therefore, it should be interpreted as a study-specific duration-based physical activity indicator rather than a standardized comprehensive physical activity questionnaire.

### Statistical analysis

All statistical analyses were performed using R software, version 4.4.1 (R Foundation for Statistical Computing, Vienna, Austria). All statistical tests were two-sided, and a P value <0.05 was considered statistically significant. Baseline characteristics of the study participants were first summarized using descriptive statistics. Normally distributed continuous variables are presented as mean ± standard deviation (SD), whereas skewed continuous variables are presented as median and interquartile range [M (Q1, Q3)]. Categorical variables are expressed as frequencies and percentages [n (%)]. Missing data were assessed before statistical analysis. Participants with missing data on key exposures, outcomes, mindfulness scores, or covariates were excluded, and complete-case analysis was performed. Because the final analytic sample consisted of 400 participants with complete data, no imputation was conducted.

The distribution of major continuous variables was assessed using the Kolmogorov–Smirnov test. Because HbA1c and fasting blood glucose (FBG) were not normally distributed, both glycemic indicators were natural log-transformed before being entered into parametric models, in order to improve the distribution of model residuals and better meet the assumptions of linear regression. Regression results are reported as β coefficients with corresponding 95% confidence intervals (CIs).

Dietary quality score and physical activity score were included as continuous variables in the primary analyses. For presentation and clinical interpretation, these scores were also categorized into lower and higher groups according to their sample distributions and analyzed as supplementary categorical variables. Multivariable linear regression models were used to examine the associations of dietary quality score and physical activity score with log-transformed HbA1c and FBG. A stepwise adjustment strategy was applied. The crude model included no covariates. Model 1 was adjusted for age and sex. Model 2 was further adjusted for educational level, smoking status, alcohol consumption, diabetes duration, body mass index (BMI), hypertension, cardiovascular disease, nighttime sleep duration, daytime nap duration, intensity of glucose-lowering medication use, and insulin use. Separate regression models were constructed for dietary quality score and physical activity score in relation to each glycemic indicator.

To evaluate the potential moderating role of trait mindfulness in the associations between lifestyle behaviors and glycemic control, multiplicative interaction terms were further constructed and incorporated into multivariable linear regression models. Specifically, the interaction terms Act with Awareness × Diet and Act with Awareness × Physical Activity were separately modeled, with HbA1c and FBG as outcome variables, to examine whether the Act with Awareness dimension of mindfulness modified the associations of dietary quality and physical activity with glycemic indicators. The statistical significance of interaction terms was assessed using Wald tests. The interaction models were adjusted for the same covariates as the fully adjusted models, including age, sex, educational level, smoking status, alcohol consumption, diabetes duration, BMI, hypertension, cardiovascular disease, nighttime sleep duration, daytime nap duration, intensity of glucose-lowering medication use, and insulin use.

The potential moderating role of trait mindfulness was primarily assessed using regression-based multiplicative interaction models rather than Structural Equation Modeling (SEM). This approach was selected because the main objective of the present study was to determine whether an observed mindfulness dimension modified the associations between observed lifestyle behavior scores and observed glycemic indicators after adjustment for covariates. The exposures, moderator, and outcomes were measured variables rather than latent constructs requiring measurement modeling. In addition, the cross-sectional design did not allow temporal ordering among lifestyle behaviors, mindfulness, and glycemic indicators to be established, which limited the appropriateness of specifying a structural pathway model. Therefore, multivariable linear regression with interaction terms was considered more consistent with the observational and association-oriented nature of this study. Stratified analyses were conducted as supplementary analyses to visualize subgroup-specific association patterns and should not be interpreted as the primary test of moderation.

Prespecified stratified analyses were conducted to further examine the consistency of the associations of dietary quality and physical activity with glycemic control across different subgroups. Stratification variables included age, sex, BMI, diabetes duration, smoking status, alcohol consumption, and mindfulness level. Age was stratified as <60 years and ≥60 years. BMI was categorized according to the Chinese adult BMI classification criteria as non-overweight (<24.0 kg/m²), overweight (24.0–27.9 kg/m²), and obesity (≥28.0 kg/m²) (21). Diabetes duration was stratified into four groups: <1 year, 1–5 years, 5–10 years, and ≥10 years, and smoking and alcohol consumption were each classified as yes or no. Total mindfulness score and scores for each mindfulness dimension were dichotomized into lower and higher levels according to the sample median. Stratified results were displayed using forest plots to visualize the direction and stability of the associations of dietary quality score and physical activity score with HbA1c and FBG across subgroups. The dichotomization of mindfulness scores was used only for stratified analyses and graphical presentation; the primary statistical inference was based on continuous-variable models and interaction analyses.

Because multiple subgroup analyses were performed, the risk of false-positive findings due to multiple testing was considered. Therefore, P values from stratified analyses were additionally adjusted using the Benjamini–Hochberg false discovery rate method within each exposure–outcome family. The FDR-adjusted results are presented in **Supplementary Tables 2**, **S3**. The primary interpretation of the study was based on the main multivariable regression models and the prespecified interaction models, whereas subgroup analyses were regarded as exploratory and hypothesis-generating.

For model diagnostics, multicollinearity was assessed using variance inflation factors (VIFs). A VIF value <5 for all covariates was considered to indicate the absence of substantial multicollinearity. Given the cross-sectional design of this study, all regression, interaction, and stratified analyses were interpreted as statistical associations rather than evidence of causality.

## Results

### Baseline characteristics

**Table 1** presents the baseline characteristics of the 400 hospitalized patients with T2DM included in this study. Overall, the participants were middle-aged, and men accounted for approximately two-thirds of the sample. The cohort showed suboptimal glycemic control, with elevated mean HbA1c and FBG levels. Regarding the main lifestyle exposures, the mean dietary quality score was 10.43 ± 1.73, and the mean physical activity score was 87.33 ± 13.02. For the psychological moderator of interest, the mean total mindfulness score was 120.02 ± 17.75, and the mean Act with Awareness score was 25.98 ± 7.06. Other demographic, lifestyle, clinical, and treatment-related characteristics, including BMI, diabetes duration, comorbidities, sleep variables, medication intensity, and insulin use, are summarized in **Table 1** and were considered as covariates in subsequent multivariable analyses. BMI and diabetes duration were retained as clinically relevant covariates because adiposity and disease duration may influence both lifestyle behaviors and glycemic control.

**Table 1:** Baseline characteristics of the study participants.

Variable	Total (n=400)
Demographic characteristics	
Age (years)	54.07 ± 11.16
Sex	
Male	252(63%)
Female	148(37%)
Education	
primary and below	63 (15.75%)
middle school	310 (77.5%)
university and above	27 (6.75%)
Lifestyle behaviors	
Smoke	
No	250 (62.5%)
Yes	150 (37.5%)
Drink	
No	298 (74.5%)
Yes	102 (25.5%)
Night sleep (hours)	6.02 ± 1.33
Nap (hours)	0.57 ± 0.65
Diet	10.43 ± 1.73
Exercise	87.33 ± 13.02
Clinical characteristics	
DM duration (years)	
<1	90 (22.5%)
1-5	94 (23.5%)
5-10	70 (17.5%)
≥10	146 (36.5%)
BMI (kg/m^2^)	26.04 ± 4.39
Hypertension	
No	224 (56%)
Yes	176 (44%)
Cardiovascular Disease	
No	356 (89%)
Yes	44 (11%)
Glucose-lowering medication intensity	
Low	141 (35.25%)
Moderate	221 (55.25%)
High	38 (9.5%)
Insulin	
No	272 (68%)
Yes	128 (32%)
Laboratory measurements	
HbA1c (%)	9.11 ± 2.37
FBG(mmol/L)	9.87 ± 3.63
TG (mmol/L)	2.32 ± 2.2
TC (mmol/L)	4.94 ± 1.28
LDL (mmol/L)	2.89 ± 0.92
HDL (mmol/L)	1.21 ± 0.31
Psychological characteristics	
Total mindfulness	120.02 ± 17.75
Observe	20.75 ± 7.62
Describe	25.33 ± 5.37
Act with Awareness	25.98 ± 7.06
Non-judging of Inner Experience	25.87 ± 5.57
Non-reactivity to Inner Experience	22.09 ± 5.68

Data are presented as mean ± standard deviation for continuous variables and n (%) for categorical variables. **Table 1** is based on the final complete-case sample of 400 participants. BMI, body mass index; DM, diabetes mellitus; HbA1c, glycated hemoglobin; FBG, fasting blood glucose; TG, triglycerides; TC, total cholesterol; LDL-C, low-density lipoprotein cholesterol; HDL-C, high-density lipoprotein cholesterol. Glucose-lowering medication intensity was categorized according to the number of non-insulin glucose-lowering medication classes used before hospital admission: low, one class; moderate, two classes; and high, three or more classes. Insulin use was recorded separately. Mindfulness was assessed using the Five Facet Mindfulness Questionnaire, including Observe, Describe, Act with Awareness, Non-judging of Inner Experience, and Non-reactivity to Inner Experience.

### Association between dietary quality and glycemic control

As shown in **Table 2**, when the dietary quality score was included as a continuous variable in multivariable linear regression models, it was inversely associated with both HbA1c and FBG. In the fully adjusted model, each 1-point increase in dietary quality score was associated with a lower log-transformed HbA1c level (β = -0.019, 95% CI: -0.032 to -0.005; P = 0.008). Similarly, a higher dietary quality score was associated with a lower log-transformed FBG level (β = -0.023, 95% CI: -0.042 to -0.003; P = 0.022). In the categorical analysis, participants in the higher dietary quality group had lower log-transformed HbA1c levels than those in the lower dietary quality group (β = -0.048, 95% CI: -0.100 to 0.004), although the difference did not reach statistical significance (P = 0.068). For FBG, the β coefficient for the higher dietary quality group compared with the lower dietary quality group was -0.063 (95% CI: -0.135 to 0.010; P = 0.092), which was also not statistically significant.

**Table 2:** Association of dietary quality with HbA1c and FBG.

Outcome	Model	Dietary quality score (continuous)		Dietary quality group (categorical)		
		β (95% CI)	P value	Reference	β (95% CI)	P value
HbA1c	Crude model	-0.016(-0.031,-0.001)	0.031	ref	-0.052(-0.107,0.002)	0.061
	Model 1	-0.017(-0.031,-0.002)	0.025	ref	-0.051(-0.105,0.004)	0.069
	Model 2	-0.019(-0.032,-0.005)	0.008	ref	-0.048(-0.100, 0.004)	0.068
FBG	Crude model	-0.02(-0.039,0.000)	0.051	ref	-0.067(-0.141,0.007)	0.076
	Model 1	-0.021(-0.040,-0.001)	0.040	ref	-0.065(-0.138,0.009)	0.085
	Model 2	-0.023(-0.042,-0.003)	0.022	ref	-0.063(-0.135, 0.010)	0.092

HbA1c and FBG were natural log-transformed before linear regression analyses. Dietary quality score was analyzed both as a continuous variable and as a categorical variable according to dietary quality groups. In the categorical analysis, the low dietary quality group was used as the reference group. Crude model was unadjusted. Model 1 was adjusted for age and sex. Model 2 was further adjusted for education level, smoking status, alcohol consumption, BMI, nighttime sleep duration, daytime nap duration, duration of T2DM, hypertension, cardiovascular disease, medication use, and insulin use. β, regression coefficient; CI, confidence interval; ref, reference; BMI, body mass index; T2DM, type 2 diabetes mellitus; HbA1c, glycated hemoglobin; FBG, fasting blood glucose.

### Interaction between mindfulness and dietary quality in relation to glycemic control

**Table 3** presents the interaction analysis of the Act with Awareness dimension of mindfulness in the association between dietary quality and glycemic indicators. In the model with log-transformed HbA1c [ln(HbA1c)] as the outcome, the interaction term Act with Awareness × dietary quality score was statistically significant (β = -0.0021, 95% CI: -0.0041 to -0.0001; P = 0.0398). This finding suggests that the association between dietary quality score and HbA1c may be more pronounced among individuals with higher levels of Act with Awareness. In other words, greater Act with Awareness may strengthen the association between healthier dietary behavior and better long-term glycemic control.

**Table 3:** Interaction effects of mindfulness and dietary quality on HbA1c and FBG.

Outcome	Variable	β (95% CI)	P value
HbA1c	Act with Awareness	0.0182 (-0.0037, 0.0408)	0.1035
	Diet	0.0363 (-0.0178, 0.0948)	0.1913
	Act with Awareness × Diet	-0.0021 (-0.0041, -0.0001)	0.0398
FBG	Act with Awareness	0.0402 (0.0095, 0.0736)	0.0108
	Diet	0.0840 (0.0074, 0.1743)	0.0322
	Act with Awareness × Diet	-0.0041 (-0.0069, -0.0014)	0.0049

HbA1c and FBG were natural log-transformed before linear regression analyses. Act with Awareness represents one dimension of the Five Facet Mindfulness Questionnaire. interaction term was constructed as Act with Awareness × Dietary quality score to examine whether Act with Awareness modified the association between dietary quality and glycemic indicators. The model was adjusted for age, sex, education level, smoking status, alcohol consumption, BMI, nighttime sleep duration, daytime nap duration, duration of T2DM, hypertension, cardiovascular disease, medication use, and insulin use. β, regression coefficient; CI, confidence interval; BMI, body mass index; T2DM, type 2 diabetes mellitus; HbA1c, glycated hemoglobin; FBG, fasting blood glucose.

In the model with log-transformed FBG [ln(FBG)] as the outcome, the interaction term Act with Awareness × dietary quality score was also statistically significant (β = -0.0041, 95% CI: -0.0069 to -0.0014; P = 0.0049). This result suggests that Act with Awareness may also play a moderating role in the association between dietary behavior and short-term glycemic control, with healthier dietary behavior being more strongly associated with lower FBG levels among individuals with higher Act with Awareness.

### Association between physical activity and glycemic control

As shown in **Table 4**, multivariable linear regression analysis indicated an inverse association between physical activity score and HbA1c. In the fully adjusted model, each 1-point increase in physical activity score was associated with a lower log-transformed HbA1c level (β = -0.002, 95% CI: -0.004 to 0.000; P = 0.034). In the categorical analysis, participants in the higher physical activity group had significantly lower log-transformed HbA1c levels than those in the lower physical activity group (β = -0.068, 95% CI: -0.125 to -0.011; P = 0.019).

**Table 4:** Association of physical activity with HbA1c and FBG.

Outcome	Model	Physical activity score (continuous)		Physical activity group (categorical)		
		β (95% CI)	P value	Reference	β (95% CI)	P value
HbA1c	Crude model	-0.003(-0.005, -0.001)	0.001	ref	-0.094(-0.152, -0.035)	0.002
	Model 1	-0.003(-0.005, -0.001)	0.002	ref	-0.086(-0.145, -0.027)	0.005
	Model 2	-0.002(-0.004, 0.000)	0.034	ref	-0.068(-0.125, -0.011)	0.019
FBG	Crude model	-0.002(-0.005, 0.000)	0.075	ref	-0.048(-0.129, 0.032)	0.238
	Model 1	-0.002(-0.005, 0.000)	0.101	ref	-0.036(-0.116, 0.045)	0.389
	Model 2	-0.001(-0.004, 0.001)	0.329	ref	-0.017(-0.097, 0.063)	0.676

HbA1c and FBG were natural log-transformed before linear regression analyses. Physical activity score was analyzed both as a continuous variable and as a categorical variable according to physical activity groups. In the categorical analysis, the low physical activity group was used as the reference group. Crude model was unadjusted. Model 1 was adjusted for age and sex. Model 2 was further adjusted for education level, smoking status, alcohol consumption, BMI, nighttime sleep duration, daytime nap duration, duration of T2DM, hypertension, cardiovascular disease, medication use, and insulin use. β, regression coefficient; CI, confidence interval; ref, reference; BMI, body mass index; T2DM, type 2 diabetes mellitus; HbA1c, glycated hemoglobin; FBG, fasting blood glucose.

In contrast, the association between physical activity score and log-transformed FBG was not statistically significant in the fully adjusted model (β = -0.001, 95% CI: -0.004 to 0.001; P = 0.329). Similarly, no significant difference in FBG was observed in the categorical analysis comparing the higher physical activity group with the lower physical activity group (β = -0.017, 95% CI: -0.097 to 0.063; P = 0.676). These findings suggest that, after multivariable adjustment, physical activity was not significantly associated with this short-term glycemic indicator.

### Interaction between mindfulness and physical activity in relation to glycemic control

**Table 5** presents the interaction analysis of the Act with Awareness dimension of mindfulness in the association between physical activity and glycemic indicators. After adjustment for age, sex, educational level, smoking status, alcohol consumption, diabetes duration, BMI, hypertension, cardiovascular disease, nighttime sleep duration, daytime nap duration, and glucose-lowering medication use, the interaction term Act with Awareness × physical activity was statistically significant in the model with log-transformed HbA1c [ln(HbA1c)] as the outcome (β = -0.0003, 95% CI: -0.0005 to -0.0001; P = 0.0175). The negative interaction coefficient suggests that, as Act with Awareness increases, the inverse association between physical activity and HbA1c may become stronger.

**Table 5:** Interaction effects of mindfulness and physical activity on HbA1c and FBG.

	*β* (95% *CI*)	*P*
HbA1c		
Act with Awareness	0.0254 (0.0009, 0.0512)	0.0427
Exercise	0.0068 (-0.0005, 0.0142)	0.0696
Act with Awareness * Exercise	-0.0003 (-0.0005, -0.0001)	0.0175
FBG		
Act with Awareness	0.0275 (-0.0074, 0.0644)	0.1233
Exercise	0.0078 (-0.0026, 0.0184)	0.1413
Act with Awareness * Exercise	-0.0004 (-0.0008, 0.0000)	0.0811

HbA1c and FBG were natural log-transformed before linear regression analyses. Act with Awareness represents one dimension of the Five Facet Mindfulness Questionnaire. The interaction term was constructed as Act with Awareness × Exercise to examine whether Act with Awareness modified the association between physical activity and glycemic indicators. The model was adjusted for age, sex, education level, smoking status, alcohol consumption, BMI, nighttime sleep duration, daytime nap duration, duration of T2DM, hypertension, cardiovascular disease, medication use, and insulin use. β, regression coefficient; CI, confidence interval; BMI, body mass index; T2DM, type 2 diabetes mellitus; HbA1c, glycated hemoglobin; FBG, fasting blood glucose.

In contrast, in the model with log-transformed FBG [ln(FBG)] as the outcome, the interaction term Act with Awareness × physical activity was not statistically significant (β = -0.0004, 95% CI: -0.0008 to 0.0000; P = 0.0811). This finding suggests that no significant moderating effect of this mindfulness dimension was observed for the association between physical activity and the short-term glycemic indicator.

### Stratified analysis of the association between dietary quality and glycemic control

**Figure 1:**
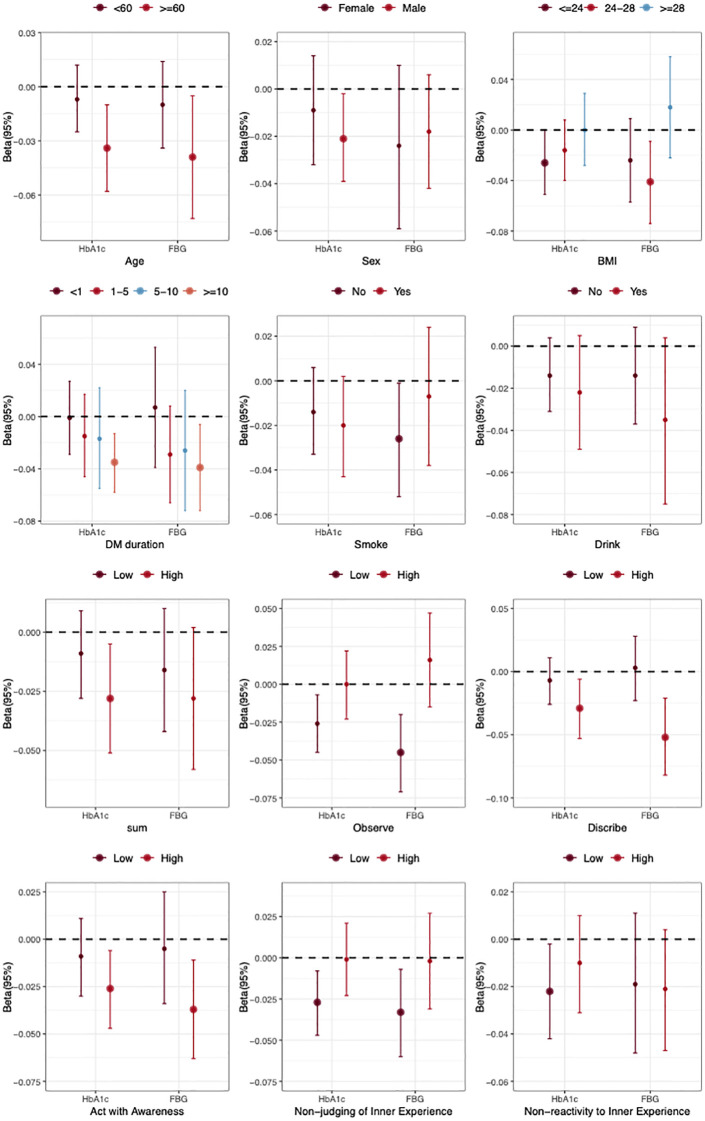
Stratified analysis of the association between dietary quality and HbA1c/FBG.

Stratified analyses showed that the associations of dietary quality score with log-transformed HbA1c and log-transformed FBG differed across participant subgroups(**Figure 1**). In the age-stratified analysis, significant inverse associations of dietary quality score with HbA1c (β = -0.034, 95% CI: -0.058 to -0.010) and FBG (β = -0.039, 95% CI: -0.073 to -0.005) were mainly observed among participants aged ≥60 years. In the sex-stratified analysis, dietary quality score was significantly inversely associated with HbA1c among men (β = -0.021, 95% CI: -0.039 to -0.002). In the BMI-stratified analysis, dietary quality score was significantly inversely associated with FBG among overweight participants with a BMI of 24–28 kg/m² (β = -0.041, 95% CI: -0.074 to -0.009). In the diabetes duration-stratified analysis, among participants with a diabetes duration of ≥10 years, dietary quality score was significantly inversely associated with both HbA1c (β = -0.037, 95% CI: -0.059 to -0.015) and FBG (β = -0.039, 95% CI: -0.072 to -0.006).

In stratified analyses by lifestyle characteristics, the inverse association between dietary quality score and HbA1c was mainly observed among participants with a history of smoking and those with alcohol consumption. For FBG, inverse associations with dietary quality score were observed across subgroups defined by smoking status and alcohol consumption. Further stratified analyses were conducted according to the median values of the total mindfulness score and each mindfulness dimension. For HbA1c, a significant inverse association between dietary quality score and HbA1c was mainly observed in the subgroup with higher total mindfulness scores (β = -0.028, 95% CI: -0.051 to -0.005; P = 0.016), whereas no statistically significant association was observed in the lower-score subgroup. Dimension-specific analyses showed that dietary quality score was significantly inversely associated with HbA1c in the higher-score subgroups for Describe and Act with Awareness. For FBG, the association between dietary quality score and FBG did not reach statistical significance in either the higher or lower total mindfulness score subgroup. However, in dimension-specific stratified analyses, significant inverse associations between dietary quality score and FBG were observed only in the higher-score subgroups for Describe and Act with Awareness, with the higher Describe subgroup showing a larger absolute β coefficient.

Given the multiple subgroup comparisons, FDR-adjusted P values were additionally calculated and are shown in **Supplementary Table 2**. After FDR correction, the inverse associations between dietary quality and ln(HbA1c) remained statistically significant in several subgroups, including older participants, men, participants with a BMI ≤24 kg/m², participants with a diabetes duration of ≥10 years, non-drinkers, and participants with higher total mindfulness or higher Act with Awareness scores. For ln(FBG), fewer subgroup associations remained statistically significant after FDR correction, mainly among participants with a diabetes duration of ≥10 years, non-smokers, and selected mindfulness subgroups. Therefore, these stratified findings were interpreted as exploratory evidence of potential heterogeneity rather than confirmatory subgroup-specific effects.

### Stratified analysis of the association between physical activity and glycemic control

**Figure 2:**
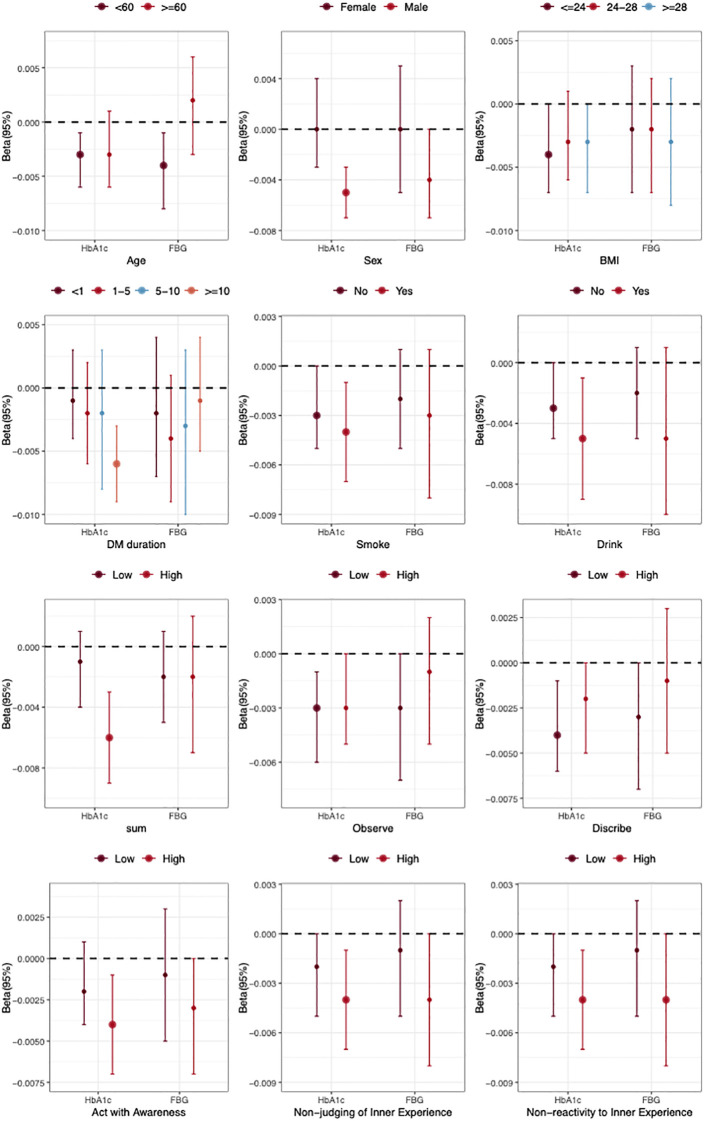
Stratified analysis of the association between physical activity and HbA1c/FBG.

Stratified analyses also showed that the associations of physical activity score with log-transformed HbA1c and log-transformed FBG varied across participant subgroups(**Figure 2**). In the age-stratified analysis, inverse associations of physical activity score with HbA1c (β = -0.003, 95% CI: -0.006 to -0.001) and FBG (β = -0.004, 95% CI: -0.008 to -0.001) were mainly observed in participants aged <60 years, whereas these associations did not reach statistical significance among those aged ≥60 years. In the sex-stratified analysis, physical activity score was inversely associated with both HbA1c (β = -0.005, 95% CI: -0.007 to -0.003) and FBG (β = -0.004, 95% CI: -0.007 to 0.000) among men. In the diabetes duration-stratified analysis, the inverse association between physical activity score and HbA1c was statistically significant among participants with a diabetes duration of ≥10 years (β = -0.006, 95% CI: -0.009 to -0.003).

Further stratified analyses were conducted according to the median values of the total mindfulness score and each mindfulness dimension. For HbA1c, the significant inverse association between physical activity score and HbA1c was mainly observed in the subgroup with higher total mindfulness scores (β = -0.006, 95% CI: -0.009 to -0.003; P < 0.001), whereas no statistically significant association was observed in the lower-score subgroup. Dimension-specific analyses showed that physical activity score was significantly inversely associated with HbA1c in the higher-score subgroups for Act with Awareness, Non-judging, and Non-reactivity, whereas these associations were not statistically significant in the corresponding lower-score subgroups. For FBG, physical activity score was not significantly associated with FBG in most subgroups. A significant inverse association was observed only in the higher Non-reactivity subgroup (β = -0.004, 95% CI: -0.008 to 0.000; P = 0.029).

FDR-adjusted P values for the stratified analyses of physical activity are presented in **Supplementary Table 3**. After FDR correction, most nominally significant subgroup associations between physical activity and ln(HbA1c) were attenuated. The inverse association remained statistically significant only among participants with a diabetes duration of ≥10 years. No subgroup association between physical activity and ln(FBG) remained statistically significant after FDR correction. These findings suggest that the subgroup patterns for physical activity should be interpreted cautiously and regarded as exploratory.

## Discussion

This study investigated the associations of dietary quality and physical activity with glycemic indicators among hospitalized patients with type 2 diabetes mellitus (T2DM), and further examined the potential moderating role of trait mindfulness in these associations. The results showed that a higher dietary quality score was associated with lower HbA1c and fasting blood glucose (FBG) levels, suggesting that patients with a healthier overall dietary pattern may have more favorable long-term and short-term glycemic status. In contrast, physical activity was inversely associated with HbA1c, whereas its association with FBG did not reach statistical significance. Further interaction analyses showed significant interactions between Act with Awareness and dietary quality for both HbA1c and FBG, as well as a significant interaction between Act with Awareness and physical activity for HbA1c. These findings suggest that the associations of diet and physical activity with glycemic control may depend not only on lifestyle behaviors themselves, but also on individual psychological self-regulatory traits.

Dietary quality was inversely associated with both HbA1c and FBG, indicating that the overall dietary pattern may play a relatively stable role in glycemic management. Dietary management is a fundamental component of T2DM treatment. Its clinical relevance lies not only in controlling total energy intake, but also in improving carbohydrate quality, increasing dietary fiber intake, optimizing fatty acid composition, and reducing the intake of foods high in sugar, fat, and salt (22). A healthier dietary pattern may be associated with better glycemic control through several pathways, including attenuation of postprandial glucose excursions, improvement in insulin sensitivity, reduction of chronic low-grade inflammation, and facilitation of weight management (22–24). In the present study, overall dietary quality was evaluated using a composite dietary score rather than by focusing on a single food group or nutrient. Therefore, the findings may more closely reflect the relationship between habitual dietary structure and glycemic status in real-world clinical settings. Notably, dietary quality score was significantly inversely associated with both HbA1c and FBG when modeled as a continuous variable, whereas comparisons between the higher and lower dietary quality groups did not fully reach statistical significance. This suggests that the relationship between dietary quality and glycemic control may be better understood as a continuous gradient, and that simple dichotomization may lead to information loss and reduced statistical power. Accordingly, the interpretation of dietary findings in this study should primarily be based on the continuous-variable models.

Physical activity was inversely associated with HbA1c but was not significantly associated with FBG, a finding that is clinically plausible. HbA1c reflects average glycemic exposure over a relatively long period and may therefore be more sensitive to the cumulative association of regular physical activity (25). Physical activity promotes skeletal muscle glucose uptake, improves insulin sensitivity, and contributes to long-term metabolic homeostasis by modulating adiposity, inflammatory status, and cardiorespiratory fitness (8, 26). Thus, the observed association between higher physical activity scores and lower HbA1c is consistent with established knowledge regarding the relationship between physical activity and glucose metabolism. In contrast, FBG is more susceptible to short-term influences, such as the previous evening meal, sleep quality, psychological stress, treatment adjustments during hospitalization, and changes in hepatic glucose production (25, 27). Therefore, in cross-sectional data, the association between physical activity and FBG may be less stable than that between physical activity and HbA1c. In addition, the physical activity score used in this study was mainly calculated according to the total weekly duration of moderate-to-vigorous physical activity, without further distinguishing activity type, intensity distribution, timing of activity, or long-term adherence. This may also have attenuated its statistical association with short-term glycemic indicators.

A particularly noteworthy finding of this study was that the Act with Awareness dimension showed significant interactions with dietary quality for both HbA1c and FBG. This result suggests that, among patients with higher levels of Act with Awareness, better dietary quality may be more strongly associated with lower glycemic indicators. Act with Awareness primarily reflects the capacity to maintain attentional focus during daily activities, engage consciously in current behavior, and reduce automatic responses (28). Dietary management does not depend solely on food choices and nutrient composition; it also involves attentional state during eating, emotional responses, recognition of hunger and satiety cues, and responses to external food cues (3, 29). Higher levels of Act with Awareness may help patients reduce distracted eating, emotional eating, and impulsive food choices, thereby making healthy dietary recommendations more likely to be translated into stable daily behaviors (30, 31). Consequently, among individuals with comparable dietary scores, those with higher levels of Act with Awareness may be more likely to demonstrate glycemic advantages associated with a healthier dietary pattern.

Similarly, the interaction between Act with Awareness and physical activity in relation to HbA1c suggests that mindfulness-related behavioral awareness may be involved in the association between physical activity and long-term glycemic control. The metabolic relevance of physical activity is not determined solely by activity duration; it may also be influenced by the quality of activity implementation, awareness of bodily sensations, activity persistence, and self-regulatory capacity (8, 32). Individuals with higher levels of Act with Awareness may be more attentive to bodily feedback, fatigue, breathing rhythm, and post-exercise glucose changes during physical activity, thereby facilitating the development of more stable and individualized activity habits (8, 13). Conversely, individuals with lower levels of Act with Awareness may engage in physical activity more passively or inconsistently, or may be less sensitive to bodily feedback, which could weaken the association between physical activity and long-term glycemic control even when a certain amount of activity is achieved (33). The absence of a significant moderating effect of Act with Awareness on the association between physical activity and FBG may be related to the greater short-term variability of FBG. It may also suggest that mindfulness-related traits are more closely linked to the long-term metabolic relevance of physical activity than to short-term fasting glucose levels.

The subgroup findings may have several possible explanations. For dietary quality, stronger inverse associations with glycemic indicators were observed among older patients and those with a longer diabetes duration. Older adults or patients with longer-standing diabetes may have more established eating habits and may be more likely to receive repeated dietary education during long-term diabetes care. In these patients, dietary quality may therefore represent a relatively stable lifestyle factor that is more closely linked to habitual glycemic status. In addition, older patients often have reduced physical capacity, more comorbidities, and greater concerns about exercise-related discomfort or hypoglycemia, which may limit the variability and intensity of physical activity. Under these circumstances, dietary structure may become a more prominent and detectable lifestyle factor in relation to glycemic control (34). This pattern may be particularly apparent for FBG because fasting glucose is sensitive to recent dietary intake, evening meal composition, nocturnal hepatic glucose production, and sleep-related metabolic changes, which may be more pronounced in older adults with longer disease duration and reduced metabolic flexibility. Longer diabetes duration may also be accompanied by greater cumulative metabolic burden, β-cell dysfunction, and insulin resistance, making sustained dietary management particularly relevant to glycemic status.

In the BMI-stratified analyses, the associations between dietary quality and glycemic indicators were less consistent. Among overweight patients, some estimates were close to zero, whereas among patients with obesity, the association with HbA1c tended to be negative but the association with FBG was unstable. These findings may reflect the complex relationship among adiposity, insulin resistance, dietary behavior, and glycemic indicators. In individuals with overweight or obesity, the glycemic relevance of dietary quality may be influenced by total energy intake, body fat distribution, medication use, insulin resistance, and unmeasured dietary components such as carbohydrate quantity, meal timing, and dietary fiber intake. Because the dietary score used in this study captured overall dietary quality rather than precise energy or macronutrient intake, it may not fully explain fasting glucose variation in patients with higher adiposity. In addition, smaller subgroup sample sizes may have contributed to wider confidence intervals and unstable estimates.

For patients with longer diabetes duration, the association between dietary quality and FBG appeared more negative than that with HbA1c, although several confidence intervals crossed zero. Clinically, this may suggest that dietary quality could be more closely related to short-term fasting glucose variation in some patients with long-standing T2DM. However, HbA1c reflects longer-term glycemic exposure and may be affected by treatment intensity, insulin use, disease progression, and cumulative metabolic impairment. Methodologically, the non-significant confidence intervals indicate that these subgroup findings should not be interpreted as definitive evidence of effect modification. Rather, they suggest possible heterogeneity that requires confirmation in larger prospective studies with validated dietary assessment tools, repeated dietary measurements, and more detailed information on energy intake, macronutrient composition, medication adjustment, and glucose monitoring.

In contrast, the associations between physical activity and glycemic indicators were more evident among younger patients and men (8, 32). Younger patients generally have better physical capacity, fewer mobility limitations, and wider variability in weekly exercise duration, which may make the relationship between physical activity and glycemic indicators easier to detect. Physical activity can improve glycemic control by enhancing skeletal muscle glucose uptake, improving insulin sensitivity, and reducing adiposity and chronic low-grade inflammation. Therefore, in subgroups with greater capacity to engage in regular activity, such as younger patients, the metabolic relevance of physical activity may be more apparent. Similarly, men in this sample may have had greater heterogeneity in exercise participation, occupational activity, smoking, alcohol consumption, and other lifestyle behaviors, which could strengthen the observed association between physical activity and glycemic control. The stronger association observed among patients with longer diabetes duration may also reflect a greater potential benefit of sustained lifestyle management in individuals with longer exposure to metabolic dysregulation.

The stronger associations observed in subgroups with higher mindfulness levels are also consistent with the interaction analyses. Patients with higher mindfulness, particularly higher Act with Awareness, may be more capable of consciously implementing dietary and physical activity recommendations, monitoring bodily feedback, and maintaining goal-directed behaviors. Therefore, lifestyle behaviors may be more closely linked to glycemic indicators in these patients. However, these interpretations remain exploratory. Because subgroup analyses were based on smaller sample sizes and involved multiple comparisons, the results should be interpreted as hypothesis-generating rather than confirmatory.

Importantly, the subgroup analyses should be interpreted with caution. Although the stratification variables were clinically relevant, multiple subgroup comparisons may increase the probability of chance findings. To address this issue, we additionally applied Benjamini–Hochberg false discovery rate correction. After correction, several subgroup associations for dietary quality, particularly in relation to HbA1c, remained statistically significant, whereas most subgroup associations for physical activity were attenuated. Therefore, these subgroup findings should be regarded as exploratory and hypothesis-generating rather than confirmatory evidence of effect modification.

From a clinical perspective, this study suggests that lifestyle management for T2DM should focus not only on whether patients are aware of dietary and physical activity recommendations, but also on whether they can implement these recommendations consistently, actively, and consciously in daily life. Conventional diabetes education often emphasizes food choices, energy control, and exercise duration, but pays relatively limited attention to attentional state, behavioral awareness, and automatic responses during eating and physical activity. The present findings showed that Act with Awareness interacted with dietary quality in relation to both HbA1c and FBG, and with physical activity in relation to HbA1c. This suggests that this dimension of mindfulness may be an important psychological trait linking lifestyle behaviors to glycemic control. Previous mindfulness-based eating interventions have emphasized awareness of hunger, satiety, emotional eating, and external food cues during eating. Their goal is not merely to increase nutritional knowledge, but to improve patients’ attentional state and self-regulatory capacity in relation to eating behavior (35, 36) Similarly, physical activity management involves not only activity duration, but also awareness of bodily sensations, fatigue, hypoglycemia risk, and post-exercise glycemic feedback (8, 33). Therefore, if confirmed in prospective or interventional studies, diabetes lifestyle management may benefit from integrating dietary and physical activity guidance with brief mindfulness-based or behavioral awareness training, such as reducing distractions during eating, attending to hunger and satiety cues, recording dietary intake and glycemic feedback, and paying attention to bodily sensations and post-exercise responses during physical activity. Such strategies should not be regarded as replacements for dietary control or physical activity management, but rather as adjunctive approaches that may improve the quality and long-term sustainability of lifestyle implementation. Importantly, because this was a cross-sectional observational study, the findings can only suggest that mindfulness-related psychological traits may be involved in the associations between lifestyle behaviors and glycemic control; they do not provide direct evidence that mindfulness-based interventions can improve glycemic control.

This study has several strengths. First, dietary quality, physical activity, and trait mindfulness were incorporated into a unified analytical framework. This allowed us not only to examine the direct associations between lifestyle behaviors and glycemic control, but also to explore the potential role of psychological self-regulatory traits in these relationships. Second, both HbA1c and FBG were used as outcome indicators, enabling the associations of lifestyle behaviors to be evaluated from the perspectives of both long-term and short-term glycemic status. Third, the multivariable models adjusted for a range of potential confounders, including demographic characteristics, disease-related factors, sleep, medication use, and insulin therapy. Stratified analyses were also performed to examine the consistency of association patterns across different patient subgroups.

Several limitations should also be acknowledged. First, the cross-sectional design means that diet, physical activity, mindfulness, and glycemic indicators were measured at the same time point. Therefore, temporal relationships cannot be established, and causal inference is not possible. Importantly, reverse causality cannot be excluded. Patients with poorer glycemic control may have recently modified their diet or physical activity after medical advice, diabetes education, or concern about elevated glucose levels. Thus, higher dietary quality or physical activity scores may partly represent behavioral responses to poor glycemic status rather than preceding lifestyle exposures, which could attenuate or obscure the observed associations. Conversely, patients with better glycemic control may have better physical function and fewer symptoms, making them more likely to maintain healthy diet and regular exercise, thereby potentially overestimating favorable lifestyle–glycemia associations. This issue may be particularly relevant for physical activity, because poorer health status, diabetes complications, or fear of hypoglycemia may limit exercise participation. Therefore, the observed associations and interaction patterns should be interpreted as cross-sectional correlations rather than evidence of temporal or causal pathways. Second, dietary quality and physical activity were assessed using investigator-developed structured scores rather than externally validated standardized instruments. Although the scoring criteria were constructed with reference to established lifestyle recommendations, including the Life’s Essential 8 framework and the Dietary Guidelines for Chinese Residents, the questionnaire has not undergone formal external validation or reproducibility testing. Therefore, measurement error, recall bias, and limited comparability with studies using validated food frequency questionnaires or physical activity questionnaires cannot be excluded. Third, the physical activity score was based only on self-reported weekly exercise duration and did not further distinguish exercise type, intensity, frequency, timing, or long-term adherence. Therefore, this score should be interpreted as a duration-based physical activity indicator rather than a comprehensive assessment of physical activity behavior. In addition, this investigator-developed score has not undergone formal external validation or reproducibility testing, which may limit its comparability with standardized physical activity questionnaires. Fourth, this study did not measure potential intermediate mechanisms that may explain the moderating role of mindfulness, such as emotional eating, eating speed, physical activity adherence, fear of hypoglycemia, stress level, cortisol, heart rate variability, insulin resistance, or postprandial glucose excursions. Finally, the study population consisted of hospitalized patients with T2DM from a single center. Hospitalized patients may have poorer glycemic control, more complex treatment regimens, and a greater disease burden than outpatient or community-based populations. Therefore, the observed associations may not be directly generalizable to patients with milder disease, more stable glycemic status, or less intensive treatment needs in outpatient or community settings.

In conclusion, among hospitalized patients with T2DM, higher dietary quality was associated with lower HbA1c and FBG levels, whereas higher physical activity was mainly associated with lower HbA1c levels. The Act with Awareness dimension of trait mindfulness may moderate the relationships of diet and physical activity with glycemic control, suggesting that psychological self-regulatory traits may be important for understanding individual differences in glycemic control related to lifestyle behaviors. Given the cross-sectional design of this study, these findings should be interpreted as observational associations. Future longitudinal cohort studies and intervention trials are needed to further verify the temporal sequence and potential mechanisms underlying these relationships.

## Conclusion

In conclusion, this cross-sectional study found that higher dietary quality was associated with lower HbA1c and fasting blood glucose levels among hospitalized patients with type 2 diabetes mellitus, whereas higher physical activity was mainly associated with lower HbA1c levels. Furthermore, the Act with Awareness dimension of trait mindfulness showed significant interactions with dietary quality in relation to both HbA1c and fasting blood glucose, and with physical activity in relation to HbA1c. These findings suggest that psychological self-regulatory traits may influence the strength of the associations between lifestyle behaviors and glycemic control. Integrating behavioral awareness or mindfulness-related strategies into conventional dietary and physical activity guidance may provide a complementary perspective for individualized diabetes management. However, given the cross-sectional design, these results should be interpreted as observational associations rather than causal relationships. Future longitudinal studies and intervention trials are needed to confirm the temporal sequence and clarify the underlying mechanisms.

## Data Availability

The raw data supporting the conclusions of this article will be made available by the authors, without undue reservation.

## References

[1] Ong KL Stafford LK McLaughlin SA Boyko EJ Vollset SE Smith AE . Global, regional, and national burden of diabetes from 1990 to 2021, with projections of prevalence to 2050: a systematic analysis for the Global Burden of Disease Study 2021. Lancet. (2023) 402:203–34. doi: 10.1016/s0140-6736(23)01301-6 37356446 PMC10364581

[2] Zheng Y Ley SH Hu FB . Global aetiology and epidemiology of type 2 diabetes mellitus and its complications. Nat Rev Endocrinol. (2018) 14:88–98. doi: 10.1038/nrendo.2017.151 29219149

[3] American Diabetes Association Professional Practice Committee for Diabetes* . 5. Facilitating positive health behaviors and well-being to improve health outcomes: Standards of care in diabetes—2026. Diabetes Care. (2025) 49:S89–S131. doi: 10.2337/dc26-S005 41358898 PMC12690188

[4] Davies MJ Aroda VR Collins BS Gabbay RA Green J Maruthur NM . Management of hyperglycemia in type 2 diabetes, 2022. A consensus report by the American Diabetes Association (ADA) and the European Association for the Study of Diabetes (EASD). Diabetes Care. (2022) 45:2753–86. doi: 10.2337/dci22-0034 36148880 PMC10008140

[5] Barrea L Vetrani C Verde L Frias-Toral E Ceriani F Cernea S . Comprehensive approach to medical nutrition therapy in patients with type 2 diabetes mellitus: From diet to bioactive compounds. Antioxidants (Basel). (2023) 12:904. doi: 10.3390/antiox12040904 37107279 PMC10135374

[6] American Diabetes Association Professional Practice Committee for Diabetes* . 8. Obesity and weight management for the prevention and treatment of diabetes: Standards of care in diabetes-2026. Diabetes Care. (2026) 49:S166–82. doi: 10.2337/dc26-S008 41358882 PMC12690172

[7] Sylow L Kleinert M Richter EA Jensen TE . Exercise-stimulated glucose uptake - regulation and implications for glycaemic control. Nat Rev Endocrinol. (2017) 13:133–48. doi: 10.1038/nrendo.2016.162 27739515

[8] Kanaley JA Colberg SR Corcoran MH Malin SK Rodriguez NR Crespo CJ . Exercise/physical activity in individuals with type 2 diabetes: A consensus statement from the American College of Sports Medicine. Med Sci Sports Exercise. (2022) 54:353–68. doi: 10.1249/MSS.0000000000002800 35029593 PMC8802999

[9] Amerkamp J Benli S Isenmann E Brinkmann C . Optimizing the lifestyle of patients with type 2 diabetes mellitus - Systematic review on the effects of combined diet-and-exercise interventions. Nutrition Metab Cardiovasc Dis. (2025) 35:103746. doi: 10.1016/j.numecd.2024.09.016 39490277

[10] Shamanna P Joshi S Thajudeen M Shah L Poon T Mohamed M . Personalized nutrition in type 2 diabetes remission: Application of digital twin technology for predictive glycemic control. Front Endocrinol (Lausanne). (2024) 15:1485464. doi: 10.3389/fendo.2024.1485464 39634180 PMC11615876

[11] Tobias DK Merino J Ahmad A Aiken C Benham JL Bodhini D . Second international consensus report on gaps and opportunities for the clinical translation of precision diabetes medicine. Nat Med. (2023) 29:2438–57. doi: 10.1038/s41591-023-02502-5 37794253 PMC10735053

[12] Favieri F Forte G Casagrande M . The executive functions in overweight and obesity: A systematic review of neuropsychological cross-sectional and longitudinal studies. Front Psychol. (2019) 10:2126. doi: 10.3389/fpsyg.2019.02126 31616340 PMC6764464

[13] Cheval B Boisgontier MP . The theory of effort minimization in physical activity. Exerc Sport Sci Rev. (2021) 49:168–78. doi: 10.1249/JES.0000000000000252 34112744 PMC8191473

[14] Creswell JD . Mindfulness interventions. Annu Rev Psychol. (2017) 68:491–516. doi: 10.1146/annurev-psych-042716-051139 27687118

[15] Goldberg SB Riordan KM Sun S Davidson RJ . The empirical status of mindfulness-based interventions: A systematic review of 44 meta-analyses of randomized controlled trials. Perspect Psychol Sci. (2022) 17:108–30. doi: 10.1177/1745691620968771 33593124 PMC8364929

[16] Tapper K . Mindfulness and craving: Effects and mechanisms. Clin Psychol Rev. (2018) 59:101–17. doi: 10.1016/j.cpr.2017.11.003 29169665

[17] Zhu D CD Society . Guideline for the prevention and treatment of type 2 diabetes mellitus in China(2020 edition). Chin J Diabetes Mellitus. (2021) 13:315–409. doi: 10.1016/b978-0-12-820472-6.00098-0 38826717

[18] Hou J Wong SYS Lo HHM Mak WWS Ma HSW . Validation of a Chinese version of the Five Facet Mindfulness Questionnaire in Hong Kong and development of a short form. Assessment. (2014) 21:363–71. doi: 10.1177/1073191113485121 23596271

[19] Wang SS Lay S Yu HN Shen SR . Dietary guidelines for Chinese residents (2016): Comments and comparisons. J Zhejiang Univ-Sci B. (2016) 17:649–56. doi: 10.1631/jzus.B1600341 27604857 PMC5018612

[20] Lloyd-Jones DM Allen NB Anderson CAM Black T Brewer LC Foraker RE . Life’s Essential 8: Updating and enhancing the American Heart Association’s construct of cardiovascular health: A presidential advisory from the American Heart Association. Circulation. (2022) 146:e18–43. doi: 10.1161/CIR.0000000000001078 35766027 PMC10503546

[21] Zhou BF Cooperative Meta-Analysis Group of the Working Group on Obesity in China . Predictive values of body mass index and waist circumference for risk factors of certain related diseases in Chinese adults--study on optimal cut-off points of body mass index and waist circumference in Chinese adults. BioMed Environ Sci. (2002) 15:83–96. doi: 10.1046/j.1440-6047.11.s8.9.x 12046553

[22] Diabetes and Nutrition Study Group (DNSG) of the European Association for the Study of Diabetes (EASD) . Evidence-based European recommendations for the dietary management of diabetes. Diabetologia. (2023) 66:965–85. doi: 10.1007/s00125-023-05894-8 37069434

[23] Guasch-Ferré M Willett WC . The Mediterranean diet and health: A comprehensive overview. J Intern Med. (2021) 290:549–66. doi: 10.1111/joim.13333 34423871

[24] Lichtenstein AHL Appel LJ Vadiveloo M Hu FB Kris-Etherton PM Rebholz CM . 2021 dietary guidance to improve cardiovascular health: A scientific statement from the American Heart Association. Circulation. (2021) 144:e472–87. doi: 10.1161/CIR.0000000000001031 34724806

[25] American Diabetes Association Professional Practice Committee . 6. Glycemic goals and hypoglycemia: Standards of care in diabetes-2024. Diabetes Care. (2024) 47:S111–25. doi: 10.2337/dc24-S006 38078586 PMC10725808

[26] Pedersen BK . The physiology of optimizing health with a focus on exercise as medicine. Annu Rev Physiol. (2019) 81:607–27. doi: 10.1146/annurev-physiol-020518-114339 30526319

[27] Vujović N Piron MJ Qian J Chellappa SL Nedeltcheva A Barr D . Late isocaloric eating increases hunger, decreases energy expenditure, and modifies metabolic pathways in adults with overweight and obesity. Cell Metab. (2022) 34:1486–1498.e7. doi: 10.1016/j.cmet.2022.09.007 36198293 PMC10184753

[28] Jiang L Zhang S Li J Gong Y Sun N Wang H . Individuals with high mindfulness are better at metacognitive ability: A latent profile analysis approach. Behav Sci (Basel). (2025) 15:1341. doi: 10.3390/bs15101341 41153131 PMC12561965

[29] Linardon J Tylka TL Fuller-Tyszkiewicz M . Intuitive eating and its psychological correlates: A meta-analysis. Int J Eat Disord. (2021) 54:1073–98. doi: 10.1002/eat.23509 33786858

[30] Yang M Wang X Zhang Y Qian W Tang Y . Mindfulness acting with awareness and emotional eating among polycystic ovary syndrome women with infertility: The mediating role of depression. Front Psychol. (2024) 15:1499705. doi: 10.3389/fpsyg.2024.1499705 39723408 PMC11669249

[31] Barnhart WR Braden AL Dial LA . Understanding the relationship between negative emotional eating and binge eating: The moderating effects of acting with awareness and non-reactive mindfulness. J Clin Psychol. (2021) 77:1954–72. doi: 10.1002/jclp.23123 33561322

[32] Gallardo-Gómez D Salazar-Martínez E Alfonso-Rosa RM Ramos-Munell J Del Pozo-Cruz J Del Pozo Cruz B . Optimal dose and type of physical activity to improve glycemic control in people diagnosed with type 2 diabetes: A systematic review and meta-analysis. Diabetes Care. (2024) 47:295–303. doi: 10.2337/dc23-0800 38241499

[33] American Diabetes Association Professional Practice Committee . 7. Diabetes technology: Standards of care in diabetes-2024. Diabetes Care. (2024) 47:S126–44. doi: 10.2337/dc24-S007 38078575 PMC10725813

[34] American Diabetes Association Professional Practice Committee . 13. Older adults: Standards of care in diabetes-2024. Diabetes Care. (2024) 47:S244–57. doi: 10.2337/dc24-S013 38078580 PMC10725804

[35] Caba U Çakir AS Turan MB Pepe O Pekel A Bahçe A . From nutrition knowledge to sustainable diets: A cross-sectional serial mediation model of dietary self-efficacy and mindful eating. Front Nutr. (2026) 13:1812781. doi: 10.3389/fnut.2026.1812781 42221786 PMC13218852

[36] Preissner CE de Ruijter D Oenema A de Vries H . Addressing individual needs in mindful eating: A latent profile analysis and exploration of demographics and social-cognitive beliefs. Health Psychol Behav Med. (2025) 13:2519587. doi: 10.1080/21642850.2025.2519587 40599682 PMC12210480

