# Supplementary material for: Associations of dietary quality and physical activity with glycemic control in patients with type 2 diabetes: the moderating role of trait mindfulness
Source: Front Endocrinol (Lausanne). 2026 Jul 22;17:1910587. doi: 10.3389/fendo.2026.1910587 (PMC13437484; doi:10.3389/fendo.2026.1910587)
Supplement: Supplementary file 1 [file Table1.docx]

**Associations of Dietary Quality and Physical Activity with Glycemic Control in Patients with Type 2 Diabetes: The Moderating Role of Trait Mindfulness**

Jiaxin Li^1^, Daowen Zhang^1^, Weiwei Zhu^1^, Huijun Zhang^1^, Cancan Liu^1^, Fang Zhang^1^, Mengmeng Yan^1^ and Kuanlu Fan^1*^

^1^ Department of Endocrinology, The Second Affiliated Hospital of Xuzhou Medical University, Xuzhou, China

*** Correspondence:**
Kuanlu Fan, MD, 32 Meijian Road, Quanshan District, Xuzhou City, Jiangsu Province, China;

telephone number:86-15962980159;

E-mail: 15962980159@163.com.

Keywords: Diet, Exercise, Type 2 diabetes, Glycemic control

**Supplementary Table S1. Components and scoring algorithm of the dietary quality score**

| **Component** | **Healthy dietary criterion** | **Score** |
| --- | --- | --- |
| Cooking oil | Met the predefined recommendation for appropriate cooking oil use | 1 = yes; 0 = no |
| Vegetables | Met the predefined recommendation for regular vegetable intake | 1 = yes; 0 = no |
| Fruits | Met the predefined recommendation for regular fruit intake | 1 = yes; 0 = no |
| Salt intake | Met the predefined recommendation for lower salt intake | 1 = yes; 0 = no |
| Red and processed meat | Met the predefined recommendation for limited red/processed meat intake | 1 = yes; 0 = no |
| Fish and seafood | Met the predefined recommendation for fish/seafood intake | 1 = yes; 0 = no |
| Poultry | Met the predefined recommendation for poultry intake | 1 = yes; 0 = no |
| Dairy products | Met the predefined recommendation for dairy intake | 1 = yes; 0 = no |
| Butter or cream | Met the predefined recommendation for limited butter/cream intake | 1 = yes; 0 = no |
| Legumes | Met the predefined recommendation for legume intake | 1 = yes; 0 = no |
| Whole grains | Met the predefined recommendation for whole-grain intake | 1 = yes; 0 = no |
| Sweets and pastries | Met the predefined recommendation for limited sweets/pastries intake | 1 = yes; 0 = no |
| Nuts | Met the predefined recommendation for nut intake | 1 = yes; 0 = no |
| Sugar-sweetened beverages | Met the predefined recommendation for limited sugar-sweetened beverage intake | 1 = yes; 0 = no |
| Fast food | Met the predefined recommendation for limited fast-food intake | 1 = yes; 0 = no |
| Alcohol intake | Met the predefined recommendation for limited or no alcohol intake | 1 = yes; 0 = no |
| **Total score** | Sum of all 16 components | **0–16** |

**Supplementary Table S2. FDR-adjusted results of stratified analyses for dietary quality and glycemic indicators**

**Panel A. Dietary quality and ln(HbA1c)**

| **Subgroup variable** | **Subgroup level** | **n** | **β (95% CI)** | **Raw P value** | **FDR-adjusted P value** |
| --- | --- | --- | --- | --- | --- |
| Age | ≥60 | 147 | -0.037 (-0.060, -0.013) | 0.003 | 0.030 |
| Age | <60 | 253 | -0.010 (-0.028, 0.007) | 0.238 | 0.338 |
| Sex | Male | 252 | -0.022 (-0.039, -0.005) | 0.013 | 0.038 |
| Sex | Female | 148 | -0.011 (-0.034, 0.011) | 0.333 | 0.409 |
| BMI | ≥28 | 108 | -0.003 (-0.031, 0.026) | 0.850 | 0.850 |
| BMI | 24-28 | 148 | -0.014 (-0.038, 0.009) | 0.231 | 0.338 |
| BMI | ≤24 | 144 | -0.032 (-0.055, -0.008) | 0.009 | 0.032 |
| Diabetes duration | 5-10 | 70 | -0.030 (-0.073, 0.013) | 0.169 | 0.269 |
| Diabetes duration | 1-5 | 94 | -0.022 (-0.051, 0.007) | 0.133 | 0.240 |
| Diabetes duration | <1 | 90 | -0.003 (-0.031, 0.025) | 0.820 | 0.850 |
| Diabetes duration | ≥10 | 146 | -0.035 (-0.059, -0.011) | 0.005 | 0.030 |
| Smoke | Yes | 150 | -0.022 (-0.042, -0.001) | 0.039 | 0.087 |
| Smoke | No | 250 | -0.019 (-0.038, -0.001) | 0.043 | 0.089 |
| Drink | No | 298 | -0.020 (-0.036, -0.003) | 0.018 | 0.043 |
| Drink | Yes | 102 | -0.021 (-0.047, 0.006) | 0.125 | 0.240 |
| Total mindfulness | Q1 | 210 | -0.009 (-0.027, 0.009) | 0.302 | 0.388 |
| Total mindfulness | Q2 | 190 | -0.031 (-0.053, -0.009) | 0.006 | 0.030 |
| Observe | Q1 | 209 | -0.032 (-0.051, -0.012) | 0.002 | 0.030 |
| Observe | Q2 | 191 | -0.004 (-0.024, 0.017) | 0.711 | 0.800 |
| Describe | Q1 | 216 | -0.010 (-0.028, 0.008) | 0.263 | 0.355 |
| Describe | Q2 | 184 | -0.029 (-0.053, -0.006) | 0.014 | 0.039 |
| Act with Awareness | Q1 | 203 | -0.008 (-0.027, 0.011) | 0.406 | 0.476 |
| Act with Awareness | Q2 | 197 | -0.027 (-0.048, -0.007) | 0.009 | 0.032 |
| Non-judging | Q1 | 224 | -0.029 (-0.049, -0.009) | 0.004 | 0.030 |
| Non-judging | Q2 | 176 | -0.003 (-0.022, 0.017) | 0.772 | 0.834 |
| Non-reactivity | Q1 | 213 | -0.025 (-0.044, -0.006) | 0.009 | 0.032 |
| Non-reactivity | Q2 | 187 | -0.014 (-0.034, 0.005) | 0.155 | 0.262 |

**Panel B. Dietary quality and ln(FBG)**

| **Subgroup variable** | **Subgroup level** | **n** | **β (95% CI)** | **Raw P value** | **FDR-adjusted P value** |
| --- | --- | --- | --- | --- | --- |
| Age | ≥60 | 147 | -0.041 (-0.075, -0.007) | 0.018 | 0.061 |
| Age | <60 | 253 | -0.014 (-0.039, 0.010) | 0.251 | 0.339 |
| Sex | Male | 252 | -0.019 (-0.042, 0.004) | 0.112 | 0.199 |
| Sex | Female | 148 | -0.032 (-0.066, 0.002) | 0.063 | 0.144 |
| BMI | ≥28 | 108 | 0.007 (-0.032, 0.046) | 0.733 | 0.825 |
| BMI | 24-28 | 148 | -0.043 (-0.076, -0.009) | 0.014 | 0.054 |
| BMI | ≤24 | 144 | -0.031 (-0.064, 0.002) | 0.067 | 0.144 |
| Diabetes duration | 5-10 | 70 | -0.032 (-0.085, 0.022) | 0.237 | 0.337 |
| Diabetes duration | 1-5 | 94 | -0.035 (-0.071, 0.001) | 0.059 | 0.144 |
| Diabetes duration | <1 | 90 | 0.013 (-0.033, 0.059) | 0.567 | 0.666 |
| Diabetes duration | ≥10 | 146 | -0.050 (-0.086, -0.013) | 0.008 | 0.038 |
| Smoke | Yes | 150 | -0.009 (-0.039, 0.021) | 0.565 | 0.666 |
| Smoke | No | 250 | -0.035 (-0.061, -0.009) | 0.008 | 0.038 |
| Drink | No | 298 | -0.021 (-0.043, 0.002) | 0.075 | 0.144 |
| Drink | Yes | 102 | -0.033 (-0.075, 0.009) | 0.126 | 0.201 |
| Total mindfulness | Q1 | 210 | -0.016 (-0.042, 0.010) | 0.221 | 0.331 |
| Total mindfulness | Q2 | 190 | -0.032 (-0.063, -0.000) | 0.050 | 0.144 |
| Observe | Q1 | 209 | -0.056 (-0.081, -0.030) | 0.000 | 0.001 |
| Observe | Q2 | 191 | 0.011 (-0.019, 0.041) | 0.477 | 0.613 |
| Describe | Q1 | 216 | 0.001 (-0.024, 0.026) | 0.945 | 0.945 |
| Describe | Q2 | 184 | -0.052 (-0.085, -0.019) | 0.002 | 0.020 |
| Act with Awareness | Q1 | 203 | -0.003 (-0.032, 0.026) | 0.827 | 0.859 |
| Act with Awareness | Q2 | 197 | -0.042 (-0.068, -0.016) | 0.002 | 0.020 |
| Non-judging | Q1 | 224 | -0.038 (-0.064, -0.011) | 0.006 | 0.038 |
| Non-judging | Q2 | 176 | -0.004 (-0.033, 0.025) | 0.780 | 0.843 |
| Non-reactivity | Q1 | 213 | -0.023 (-0.053, 0.006) | 0.118 | 0.199 |
| Non-reactivity | Q2 | 187 | -0.023 (-0.049, 0.002) | 0.072 | 0.144 |

Note: HbA1c and FBG were natural log-transformed before regression analyses. β coefficients and 95% CIs represent the estimated change in ln(HbA1c) or ln(FBG) associated with each 1-point increase in the dietary quality score within each subgroup. Multivariable linear regression models were adjusted for age, sex, education level, smoking status, alcohol consumption, BMI, nighttime sleep duration, daytime nap duration, duration of T2DM, hypertension, cardiovascular disease, medication use, and insulin use, except for the corresponding stratification variable when applicable. Raw P values refer to nominal P values before correction. FDR-adjusted P values were calculated using the Benjamini–Hochberg method within each exposure–outcome family. Q1 and Q2 indicate lower and higher levels of mindfulness scores divided according to the sample median, respectively. FDR, false discovery rate; CI, confidence interval; BMI, body mass index; T2DM, type 2 diabetes mellitus; HbA1c, glycated hemoglobin; FBG, fasting blood glucose.

**Supplementary Table S3. FDR-adjusted results of stratified analyses for physical activity and glycemic indicators**

**Panel A. Physical activity and ln(HbA1c)**

| **Subgroup variable** | **Subgroup level** | **n** | **β (95% CI)** | **Raw P value** | **FDR-adjusted P value** |
| --- | --- | --- | --- | --- | --- |
| Age | ≥60 | 147 | -0.002 (-0.006, 0.001) | 0.203 | 0.342 |
| Age | <60 | 253 | -0.002 (-0.004, 0.001) | 0.154 | 0.298 |
| Sex | Male | 252 | -0.003 (-0.006, -0.001) | 0.004 | 0.060 |
| Sex | Female | 148 | 0.001 (-0.002, 0.004) | 0.410 | 0.563 |
| BMI | ≥28 | 108 | -0.004 (-0.007, -0.000) | 0.030 | 0.164 |
| BMI | 24-28 | 148 | -0.000 (-0.003, 0.003) | 0.873 | 0.907 |
| BMI | ≤24 | 144 | -0.003 (-0.007, 0.000) | 0.058 | 0.186 |
| Diabetes duration | 5-10 | 70 | -0.002 (-0.008, 0.004) | 0.457 | 0.563 |
| Diabetes duration | 1-5 | 94 | -0.002 (-0.005, 0.002) | 0.426 | 0.563 |
| Diabetes duration | <1 | 90 | 0.001 (-0.003, 0.004) | 0.727 | 0.785 |
| Diabetes duration | ≥10 | 146 | -0.005 (-0.008, -0.002) | 0.002 | 0.043 |
| Smoke | Yes | 150 | -0.003 (-0.006, 0.000) | 0.081 | 0.220 |
| Smoke | No | 250 | -0.002 (-0.004, 0.001) | 0.190 | 0.342 |
| Drink | No | 298 | -0.002 (-0.004, 0.000) | 0.125 | 0.259 |
| Drink | Yes | 102 | -0.004 (-0.007, 0.000) | 0.051 | 0.186 |
| Total mindfulness | Q1 | 210 | -0.001 (-0.003, 0.002) | 0.550 | 0.638 |
| Total mindfulness | Q2 | 190 | -0.004 (-0.008, -0.001) | 0.015 | 0.102 |
| Observe | Q1 | 209 | -0.003 (-0.005, -0.000) | 0.049 | 0.186 |
| Observe | Q2 | 191 | -0.001 (-0.004, 0.001) | 0.324 | 0.485 |
| Describe | Q1 | 216 | -0.002 (-0.005, 0.000) | 0.062 | 0.186 |
| Describe | Q2 | 184 | -0.001 (-0.004, 0.002) | 0.458 | 0.563 |
| Act with Awareness | Q1 | 203 | -0.000 (-0.003, 0.003) | 0.972 | 0.972 |
| Act with Awareness | Q2 | 197 | -0.003 (-0.006, -0.001) | 0.015 | 0.102 |
| Non-judging | Q1 | 224 | -0.002 (-0.005, 0.000) | 0.105 | 0.257 |
| Non-judging | Q2 | 176 | -0.001 (-0.004, 0.002) | 0.567 | 0.638 |
| Non-reactivity | Q1 | 213 | -0.002 (-0.004, 0.000) | 0.122 | 0.259 |
| Non-reactivity | Q2 | 187 | -0.002 (-0.005, 0.001) | 0.230 | 0.365 |

**Panel B. Physical activity and ln(FBG)**

| **Subgroup variable** | **Subgroup level** | **n** | **β (95% CI)** | **Raw P value** | **FDR-adjusted P value** |
| --- | --- | --- | --- | --- | --- |
| Age | ≥60 | 147 | 0.002 (-0.003, 0.007) | 0.435 | 0.903 |
| Age | <60 | 253 | -0.003 (-0.006, 0.000) | 0.085 | 0.802 |
| Sex | Male | 252 | -0.002 (-0.005, 0.001) | 0.157 | 0.802 |
| Sex | Female | 148 | 0.001 (-0.004, 0.005) | 0.720 | 0.903 |
| BMI | ≥28 | 108 | -0.003 (-0.008, 0.002) | 0.202 | 0.802 |
| BMI | 24-28 | 148 | -0.001 (-0.005, 0.004) | 0.806 | 0.903 |
| BMI | ≤24 | 144 | -0.002 (-0.006, 0.003) | 0.523 | 0.903 |
| Diabetes duration | 5-10 | 70 | -0.004 (-0.011, 0.004) | 0.346 | 0.903 |
| Diabetes duration | 1-5 | 94 | -0.003 (-0.008, 0.002) | 0.253 | 0.802 |
| Diabetes duration | <1 | 90 | -0.001 (-0.007, 0.005) | 0.751 | 0.903 |
| Diabetes duration | ≥10 | 146 | -0.000 (-0.005, 0.005) | 0.922 | 0.922 |
| Smoke | Yes | 150 | -0.002 (-0.006, 0.003) | 0.445 | 0.903 |
| Smoke | No | 250 | -0.001 (-0.004, 0.002) | 0.573 | 0.903 |
| Drink | No | 298 | -0.001 (-0.004, 0.002) | 0.662 | 0.903 |
| Drink | Yes | 102 | -0.003 (-0.009, 0.003) | 0.267 | 0.802 |
| Total mindfulness | Q1 | 210 | -0.001 (-0.004, 0.002) | 0.600 | 0.903 |
| Total mindfulness | Q2 | 190 | -0.001 (-0.006, 0.004) | 0.701 | 0.903 |
| Observe | Q1 | 209 | -0.003 (-0.006, 0.001) | 0.157 | 0.802 |
| Observe | Q2 | 191 | -0.001 (-0.004, 0.003) | 0.772 | 0.903 |
| Describe | Q1 | 216 | -0.002 (-0.006, 0.001) | 0.236 | 0.802 |
| Describe | Q2 | 184 | -0.000 (-0.004, 0.004) | 0.907 | 0.922 |
| Act with Awareness | Q1 | 203 | 0.001 (-0.003, 0.005) | 0.743 | 0.903 |
| Act with Awareness | Q2 | 197 | -0.002 (-0.006, 0.001) | 0.183 | 0.802 |
| Non-judging | Q1 | 224 | -0.001 (-0.004, 0.003) | 0.626 | 0.903 |
| Non-judging | Q2 | 176 | -0.002 (-0.006, 0.002) | 0.449 | 0.903 |
| Non-reactivity | Q1 | 213 | -0.000 (-0.004, 0.003) | 0.836 | 0.903 |
| Non-reactivity | Q2 | 187 | -0.003 (-0.007, 0.001) | 0.169 | 0.802 |

Note: HbA1c and FBG were natural log-transformed before regression analyses. β coefficients and 95% CIs represent the estimated change in ln(HbA1c) or ln(FBG) associated with each 1-point increase in the physical activity score within each subgroup. Multivariable linear regression models were adjusted for age, sex, education level, smoking status, alcohol consumption, BMI, nighttime sleep duration, daytime nap duration, duration of T2DM, hypertension, cardiovascular disease, medication use, and insulin use, except for the corresponding stratification variable when applicable. Raw P values refer to nominal P values before correction. FDR-adjusted P values were calculated using the Benjamini–Hochberg method within each exposure–outcome family. Q1 and Q2 indicate lower and higher levels of mindfulness scores divided according to the sample median, respectively. FDR, false discovery rate; CI, confidence interval; BMI, body mass index; T2DM, type 2 diabetes mellitus; HbA1c, glycated hemoglobin; FBG, fasting blood glucose.
